# The role of glutamate receptors in the regulation of the tumor microenvironment

**DOI:** 10.3389/fimmu.2023.1123841

**Published:** 2023-02-01

**Authors:** Stephane Koda, Jing Hu, Xiaoman Ju, Guowei Sun, Simin Shao, Ren-Xian Tang, Kui-Yang Zheng, Juming Yan

**Affiliations:** ^1^ Jiangsu Key Laboratory of Immunity and Metabolism, Department of Pathogenic Biology and Immunology, National Experimental Demonstration Center for Basic Medicine Education, Xuzhou Laboratory of Infection and Immunity, Xuzhou Medical University, Xuzhou, Jiangsu, China; ^2^ Department of Bioinformatics, School of Life Science, Xuzhou Medical University, Xuzhou, Jiangsu, China; ^3^ Department of Genetics, Xuzhou Medical University, Xuzhou, Jiangsu, China

**Keywords:** glutamate receptors, glutamate, tumor micro-environment, immunoregulation, metabolic processes

## Abstract

Glutamate, as one of the most important carbon sources in the TCA cycle, is central in metabolic processes that will subsequently influence tumor progression. Several factors can affect the expression of glutamate receptors, playing either a tumor-promoting or tumor-suppressor role in cancer. Thus, the activation of glutamate receptors by the ligand could play a role in tumor development as ample studies have demonstrated the expression of glutamate receptors in a broad range of tumor cells. Glutamate and its receptors are involved in the regulation of different immune cells’ development and function, as suggested by the receptor expression in immune cells. The activation of glutamate receptors can enhance the effectiveness of the effector’s T cells, or decrease the cytokine production in immunosuppressive myeloid-derived suppressor cells, increasing the antitumor immune response. These receptors are essential for the interaction between tumor and immune cells within the tumor microenvironment (TME) and the regulation of antitumor immune responses. Although the role of glutamate in the TCA cycle has been well studied, few studies have deeply investigated the role of glutamate receptors in the regulation of cancer and immune cells within the TME. Here, by a systematic review of the available data, we will critically assess the physiopathological relevance of glutamate receptors in the regulation of cancer and immune cells in the TME and provide some unifying hypotheses for futures research on the role of glutamate receptors in the immune modulation of the tumor.

## 1 Introduction

**Figure 1:**
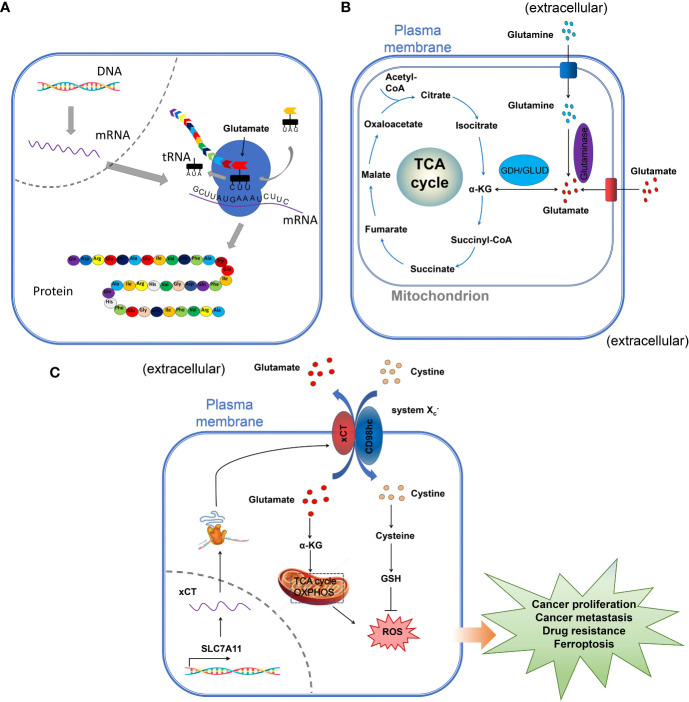
The role of glutamate in cells. **(A)** the role of glutamate in protein synthesis. The amino-acid glutamate is the substrate for protein synthesis, important for the maintenance and promotion of cell function. **(B)** the role of glutamate in the TCA. Extracellular glutamate is one of the most important sources of carbon in The TCA cycle. In the mitochondria, glutamate is deaminated to α-ketoglutarate (α-KG) by glutamate dehydrogenase (GDH), which is incorporated in the TCA cycle for energy production. **(C)** glutamate and the maintenance of cell homeostasis and regulation of cell proliferation. The amino-acid exchangers also known as cystine-glutamate transporter (xCT/SLC7A11), play a very important role in maintaining redox homeostasis by exchanging extracellular cystine in exchange for intracellular glutamate to maintain intracellular redox and the energy production for the regulation of cell proliferation.

Glutamate (glu), a non-essential amino acid involved in many metabolism processes, is one of the most important carbon sources in the TCA cycle. Glutamate can be generated by the amino-acid glutamine by the mitochondrial glutaminase (GLS). Glutamate dehydrogenase (GDH) and aspartate aminotransferase (AAT) catalyze the reversible reaction between glutamate and α-ketoglutarate (α-KG) before the incorporation of glutamate into the TCA cycle (1, 2). Glutamate can also serve as a building block for the biosynthesis of glutathione (GSH), a tripeptide composed of cysteine, glycine, and γ-linked glutamate (**Figure 1A**) (3, 4), important in the maintenance of redox homeostasis to protect the cell from oxidative damage. Additionally, glutamate is the major neurotransmitter involved in the communication with effector cells in the nervous system (5). In early 2000, the role of glutamate receptors (GluRs) in the regulation of proliferation, migration, and survival of neuronal progenitor cells was shown (6). Glutamate can activate two different types of receptors: ionotropic receptors (iGluRs), and metabotropic glutamate receptors (mGluRs).

Compared to normal cells, cancer cells are characterized by the rapid proliferation of cells, followed by a highly active metabolism (7). Glutamate is crucial in cancer and rapidly proliferating cells by directly participating in the abovementioned metabolic processes (8). Glutamate also influences tumor progression by acting on its receptors as ample studies have demonstrated the expression of glutamate receptors in a broad range of tumor cells (9, 10). The role of glutamate receptors was proved on the basis of their influence on tumor cell proliferation, survival, invasion, etc., by the use of selective antagonists and agonists in addition to genetically modulating the receptor expression in tumor cells.

### 1.1 Glutamate receptors and tumor microenvironment

Cancer cells with different types of cells such as endothelial cells, fibroblasts, immune cells, and extracellular components (11) form a specific type of environment called a tumor microenvironment (TME) (12). The intense metabolic activity of cancer cells can profoundly change the nutrient composition and the structure of the cells and tissues constituting the TME. The glutamate and GluRs are important for the interaction between various cells and the regulation of tumor growth. Because of the hypoxic conditions and the depleted nutrients in the TME, cancer cells and immune cells can enter into competition for the available nutrient, which can significantly reduce the effectiveness of the effector immune cells (13). As components of the TME, infiltrated innate [macrophages, mast cells, neutrophils, dendritic cells (DCs), myeloid-derived suppressor cells (MDSCs), and natural killer (NK) cells] and adaptive immune cells (T and B lymphocytes) within the TME are also crucial in the regulation of tumor growth (12). Glutamate receptors are involved in the regulation of different immune cells’ development and function as suggested by the receptor expression in immune cells (14, 15).

The role of glutamate in the regulation of the metabolism in the TCA cycle has been amply investigated previously and reviewed (**Figure 1B**) [See references (16, 17)] (**Figure 1** summarizes the different roles of glutamate in the cell). Also, the crucial role played by glutamate in the maintenance of redox homeostasis by exchanging extracellular cystine in exchange for intracellular glutamate through the cystine-glutamate transporter (xCT/SLC7A11) was investigated in cancer (**Figure 1C**) (18). Moreover, the role of glutamate as a neurotransmitter in the central nervous system has been well studied. As a cell surface receptor, glutamate receptors might be involved in the regulation of different cells including cancer and immune cells in the TME, however, few studies have deeply investigated this function of glutamate receptors. In this paper, by a systematic review of the available data, we will critically assess the physiopathological relevance of glutamate receptors in the regulation of cancer and immune cells in the TME and provide some unifying hypotheses for futures research on the role of glutamate receptors in the immuno-modulation of the tumor.

## 2 Generalities in glutamate receptors

Glutamate receptors are divided into two main groups based on the mechanisms involved in the reception of the signals from glutaminergic neurons and the mechanism by which they relay their signal: the metabotropic glutamate receptors (mGluRs) and the ionotropic glutamate receptors (iGluRs) (19–21).

### 2.1 Metabotropic glutamate receptors (mGluRs)

Metabotropic glutamate receptors belong to the family C G-protein-coupled receptors (GPCR), the largest and most diverse group of membrane receptors in eukaryotes (19). Various extracellular ligands such as glutamate (the natural ligand of mGluRs) and other synthetics agonists can activate mGluRs and the transduction of the signal occur through the interaction with the G-protein changing the conformation of GPCR (19). The G-protein is composed of three different units called heterotrimeric complexes of α, β, and γ subunits. The extracellular part of the mGluRs contains a large binding site for glutamate (19). mGluRs are involved in a large variety of cellular activities, including cell proliferation, metabolic reprogramming, upregulation of the immune response, and communication in the central nervous system (CNS). The mGluRs are composed of eight groups, divided into three sub-groups:

Group I comprised mGluR1 and mGluR5, which are coupled to phospholipase C (PLC) (22). These receptors are primarily expressed in neuronal cells such as glial cells and non-neuronal cells. Group II consists of mGluR2 and mGluR3 (22) and is negatively coupled to adenylate cyclase (AC). Group II mGluRs are expressed in glioblastoma cells where the antagonist suppressed the cell growth (23). In non-neuronal cells, group II mGluRs have been implicated in several types of cancers. In melanoma, for example, hot spots in mGluR3 that regulate MEK are frequently mutated resulting in the growth of cells and migration (24). Group III encompasses mGluR4, mGluR6, mGluR7, and mGluR8 and is also negatively coupled to adenylate cyclase (22). This group mGluRs have also been implicated in various cancer including colorectal, laryngeal squamous cell, breast cancers, osteosarcoma, and melanoma (25).

### 2.2 Ionotropic glutamate receptors (iGluRs)

The iGluRs are ligand-gated cation channels. According to the synthetic agonist that activates them, three subtypes of iGluRs have been described so far: *N*-methyl-D-aspartate (NMDA), α-amino-3-hydroxy-5-methyl-4-isoxazole propionic acid (AMPA), and 2-carboxy-3-carboxymethyl-4-isopropenyl pyrrolidine (kainate, KA) receptors (20, 26), which primarily mediate the signal transmission at neuronal synapses, where they contribute centrally to the postsynaptic plasticity. These receptors have also been involved in the regulation of different tumor growth.

**Figure 2:**
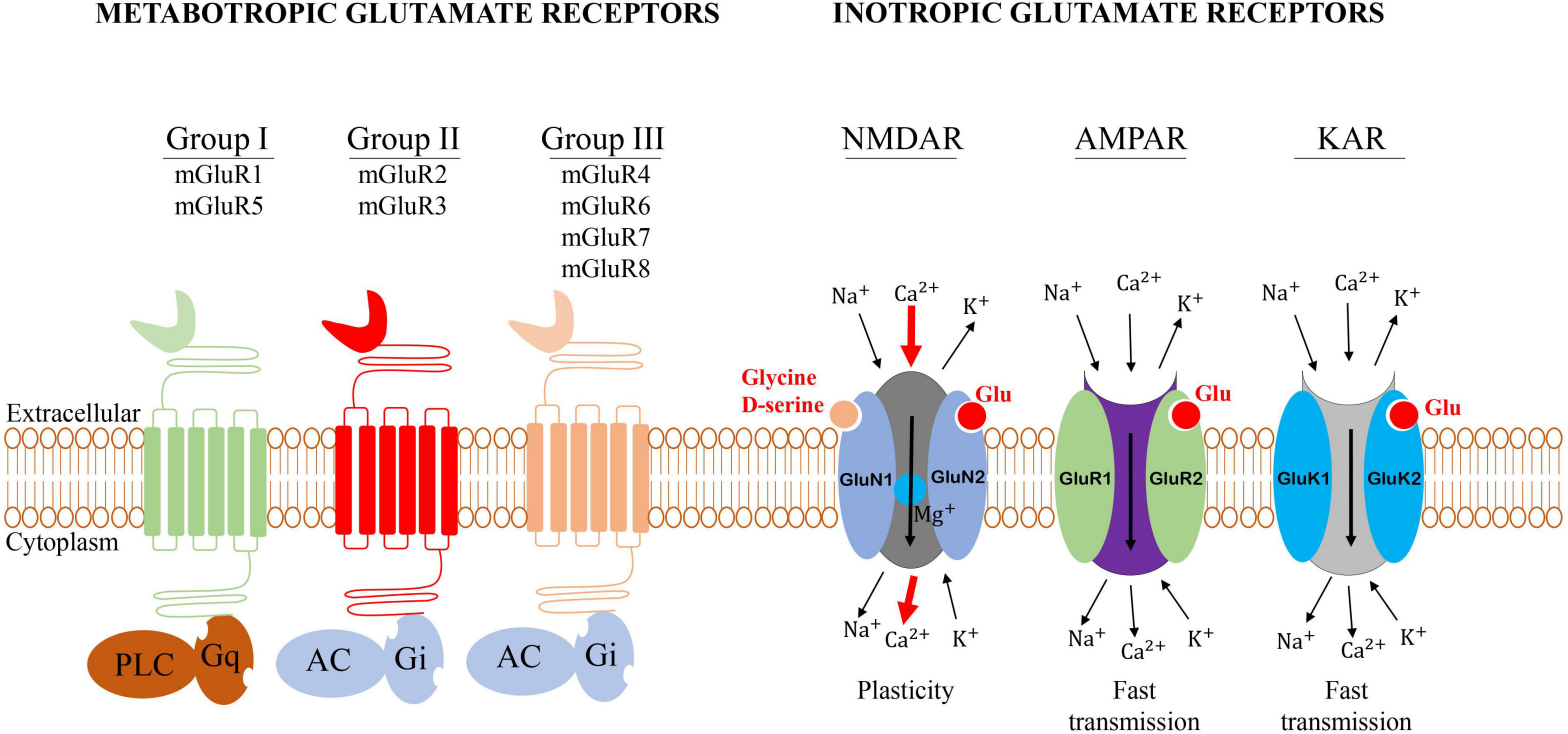
General structure of glutamate receptors. Metabotropic glutamate receptors belong to the family C G-protein-coupled receptors (GPCR). Group I mGluRs is coupled to phospholipase C, while groups II and III are negatively coupled to adenylate cyclase (AC). The iGluRs are ligand-gated cation channels that are divided into three groups: *N*-methyl-D-aspartate (NMDA), α-amino-3-hydroxy-5-methyl-4-isoxazole propionic acid (AMPA), and 2-carboxy-3-carboxymethyl-4-isopropenyl pyrrolidine (kainate, KA) receptors.

The NMDA receptor (NMDAR) is an assembly of four different units called heterotetramer, of which two obligatory glycine-binding GluN1 subunits and two glutamate-binding GluN2A-D subunits (27, 28). NMDAR is a complex macromolecular entity with different regulatory sites (26). Under normal physiological conditions, the NMDAR is quiescent, however, a strong and repeated membrane depolarization can activate the NMDAR (26). L-glutamate and D-aspartate, which are the natural ligands of NMDAR are required for their activation, thus acting as the first messenger (29). The activation of these receptors is voltage-dependent and is regulated by polyamines such as Zn^2+^ and Mg^2+^ ions (30). The Mg^2+^ ions, in particular, played an important role in the blockage of the NMDAR in neurons, thus, neurons depolarization can relieve the Mg^2+^ block and open the NMDAR channel. As ligand-gated ion channels, the NMDAR is permeable to Na^+^, K^+^, and Ca^2+^ which acts as a secondary messenger, triggering a postsynaptic response resulting in several biological processes (**Figure 2**) (31).

The AMPA receptor (AMPAR) is composed of four subunits known as GluR1-GluR4. Upon binding with the agonist, the depolarization of the membrane induces the permeability to Na^+^/Ca^2+^ ions triggering a fast excitatory postsynaptic response in neurons (5). The properties of the channel and the transmission of the electric stimulation depend on the subunit composition. Thus, the permeability of the membrane to Ca^2+^ is determined by the GluR2 subunit which plays a very important role in this process (5). The AMPAR mediates fast excitatory synaptic signaling in the brain and is involved in the activity-dependent modulation of synaptic plasticity (**Figure 2**) (32).

The KA receptor is functionally similar to the AMPAR. KA receptor is composed of GluR5–7 and KA1/2 subunits forming a tetrameric channel (**Figure 2**) (5). Very few studies have investigated the role of KA receptor in tumors therefore in this review, we will focus on NMDA and AMPA receptors.

## 3 Glutamate transporters/exchangers and regulation of glutamatergic signaling in the TME

The maintenance of the balance between the extracellular and the intracellular glutamate is made by transporters, that are expressed by tumor and immune cells. The Solute Carrier 1A (SLC1A) family includes the alanine serine cysteine transporters (ASCT) and the human glutamate transporters known as the excitatory amino acid transporters (EAAT) (33).

### 3.1 Glutamate transporters/exchangers and cancer

Two subfamilies of ASCT have been described so far, the ASCT1 (SLC1A4), and ASCT2 (SLC1A5). The ASCT is involved in the transportation of neutral-amino acids such as alanine, serine, cysteine, threonine, and valine, but also L-asparagine and L-glutamine especially for ASCT2 (SLC1A5). However, the EAATs include EAAT1 (SLC1A3), EAAT2 (SLC1A2), EAAT3 (SLC1A1), EAAT4 (SLC1A6), and EAAT5 (SLC1A7) can use aspartate and glutamate as substrates (33, 34). Another important transporter is the amino-acid exchanger also known as cystine-glutamate transporter (xCT/SLC7A11), which plays a very important role in maintaining redox homeostasis by exchanging extracellular cystine in exchange for intracellular glutamate to maintain intracellular redox state Cl^-^ dependent, Na^+^-independent manner (**Figure 1C**) (18). xCT is expressed in several types of cancer, including melanoma (35), bladder cancer (36), and triple-negative breast cancer (37). In glioma, the increase in glutamate concentration induces the influx of Ca^2+^ followed by a decrease in cell viability (38). EAATs and xCT cooperate to ensure a proper physiological function of the cells. The role of these transporters and exchangers depends on the type of cancer cells. Thus, in brain cancer, neoplastic transformation of glioma and glioblastoma cells decreases EAAT-mediated glutamate uptake (EAAT1 and EAAT2), with an increase in cystine efflux by the xCT, leading to the increase in synaptic glutamate concentration and the neurodegeneration of the surrounding neuronal cell due to the high concentration of glutamate (39). The degenerescence of the normal cells is associated with an increase in the growth of cancer cells. Interestingly, the upregulation of EAAT2 in glioma induces apoptosis and reduces the growth of tumor cells (40). Therefore, the upregulation of EAATs in brain cancers such as glioma and glioblastoma might be favorable to increasing glutamate uptake in the intracellular milieu (39).

In non-neuronal cancer cells, in contrast, the upregulation of the expression of EAATs can promote tumor growth and invasion. In choroid plexus cancer, EAAT1 is highly expressed and can be used as a marker for this type of cancer (41). Also, different from neuronal cancer (glioma), the overexpression of EAATs in non-neuronal cancer is correlated with the increase in cell growth and survival leading to the progression of the tumor by the increase in glutamate concentration (42). Glutamate is an extracellular ligand that can activate different glutamate receptors in an autocrine/paracrine loop, activating signaling pathways involved in cell proliferation (43). It was reported that upon binding of glutamate, mGluR3 undergoes glutamate-dependent rapid desensitization, internalization, trafficking, and recycling which was coupled to β-arrestin, opening the possibility of signaling to various effectors, including ERK, AKT, and JNK that can regulate cell cycle (44). Also, glutamate is an important source of carbon in the TCA cycle, playing an important role in cell metabolism. In breast cancer, the increase in cell proliferation was associated with the increase in the uptake of glutamate and aspartate as a substrate for the TCA cycle increasing the metabolism. The increase in glutamate and aspartate in breast cancer is mediated by EAAT2 (42). The increased expression of EAAT1 has also been shown to increase the uptake of aspartate in breast, colon, and lung cancer allowing the growth of tumor cells in hypoxic conditions (45).

### 3.2 Glutamate transporters/exchangers and immune cells

EAATs are crucial for the transport of glutamine, which is indispensable for the activation of T cells. The activation of T cells with CD3 and CD28 increases EAATs expression levels and increases glutamine uptake by T cells (46, 47). Similarly, it has been demonstrated that TCR-stimulated naive CD4^+^ T cells enhance the uptake of glutamine through an increase in SLC1A5 expression (46).

The treatment of mice with xCT inhibitor or the genetic deletion of xCT significantly inhibited melanoma cell proliferation, however, the combination of xCT inhibition and anti-PD-1/PD-L1 therapy reduced the efficacy of anti-PD-1/anti-PD-L1 treatment in melanoma by impairing the cytotoxicity of CD8^+^ T cells and inducing M2 macrophage polarization. The explanation of the effects of xCT inhibition on the efficacy of anti-PD-1/anti-PD-L1 treatment is unknown, but it’s possible that, the inhibition of xCT could alter the concentration of glutamate and cystine, which might partially influence the efficacy of immune checkpoint blockade (48). Recently, the high expression of xCT in (TAM) suggested that xCT may be involved in the regulation of the TAM in the tumor microenvironment (49). Interestingly, the high expression of xCT in lung cancer patients was associated with poor prognosis, and xCT expression was negatively correlated with TAM invasion, suggesting that xCT promotes tumor growth by regulating TAM. The deletion of xCT in macrophages did not induce a significant difference in the infiltration of macrophages but significantly reduced the proportion of M2-type polarized macrophages by the downregulation of the AKT/STAT6 signaling pathway while increasing the population of CD8^+^ T cells (49).

### 3.3 Role of Riluzole in the blockage of glutamate secretion

Glutamate transporters and exchangers are important in the regulation of the intra- and extracellular glutamate concentration. The availability of glutamate inside the cell contributes to the activation of glutamate receptors followed by inducing different signals leading to the regulation of cell growth. Several studies have demonstrated that the inhibition of glutamate uptake can decrease tumor growth. Riluzole, [2−amino 6 (trifluoromethoxy)benzo−thiazole] is a drug that was approved by the FDA due to its neuroprotective properties (50). The mechanism of Riluzole is not fully understood, however, it has been demonstrated that Riluzole blocks glutamate release and enhances glutamate reuptake, resulting in the inhibition of glutamate−dependent signaling (51). This drug is of importance in diseases where glutamate plays an important role, such as cancer. In glioma, for instance, the silencing of xCT by small interfering RNA decreased neuro degenerescence by the decrease in glutamate concentration (52). Thus, the use of Riluzole in glioma could be a benefit for decreasing tumor growth. The reduced tumor growth by Riluzole was confirmed in GRM3−expressing glioma in xenograft mice (53). Similarly, the proliferation of melanoma cell in xenograft−bearing animals was shown to be decreased by Riluzole probably by the inhibition of xCT (35). It’s therefore reasonable to hypothesize that the Riluzole may act by the blockage of xCT and other glutamate transporters that could subsequently decrease or inhibit the expression of glutamate receptors and decrease tumor growth.

In cancer, glutamate receptor overexpression is linked with the activation of different signaling pathways including PI3K/AKT/mTOR, Ras−MAPK−ERK, and MAPK/ERK, which are involved in the regulation of cell proliferation, survival leading to tumorigenesis (43, 54, 55). The use of Riluzole was shown to decrease MAPK/ERK and PI3K/AKT pathways hyperactivity in melanoma (56). The efficacity of the mTOR inhibitor was also enhanced by the combination with Riluzole (57). In addition to its role in the blockage of glutamate, Riluzole has been associated with the inhibition of spontaneous Ca^2+^ signaling in the immortalized growth hormone−secreting pituitary cell line GH3 (58). This function of Riluzole is important, especially for the regulation of inotropic glutamate receptors whose activation induces Ca^2+^ influx. All these data together agree with the hypothesis that Riluzole could be used as a potential therapeutic target for the modulation of glutamate receptors.

## 4 Role of glutamate receptors in cancer

### 4.1 The involvement of metabotropic glutamate receptor (mGluR) in cancer

In this section, we will highlight the pathophysiological role of glutamate receptors in cancer.

#### 4.1.1 Group I mGluRs and cancer

The group I mGluRs was reported to be involved in different types of cancer. The expression of mGluR1 has been shown in human glioma U87 cells (59), human breast cancer cells MDA-MB-231 (60), and mouse B16F10 melanoma cells (61). Similarly, the expression of mGluR5 was detected in glioma, melanoma, oral squamous cell carcinoma (SCC) tissues, and oral cancer cell lines (62) among others.

##### 4.1.1.1 mGluR1 and glioma

In glioma, the silence of mGluR1 by a specific targeted small interfering RNA (siRNA) or the inhibition by an allosteric selective antagonist BAY36-7620 (50 μM) and a non-selective antagonist Riluzole (50 μM) of mGluR1, induced the apoptosis of U87 glioma cells. The inhibition of mGluR1 decreased the invasion of U87 cells at a comparable level (53-60%) (59). Interestingly, the inhibition of mGluR1 signaling was associated with the downregulation of the PI3K/AKT/mTOR signaling pathway, indicating that mGluR1 affects glioma *via* this signaling pathway (59). The use of the mGluR1 agonist L-quisqualic acid reverses the antitumor effects of mGluR1 antagonists (BAY36-7620 and Riluzole) shown by the increase in cell proliferation and invasion, and the increase in tumor growth *in vivo*. This supported the tumor-promoting role of mGluR1. It is important to mention that, L-quisqualic acid targets different receptors including mGluR1, AMPAR, and Kainate receptor, therefore, the interaction of L-quisqualic acid with AMPAR and Kainate cannot be excluded. Thus, the use of a specific mGluR1 agonist could be an important element to consider to confirm the highlighted signaling pathways linking mGluR1 and glioma (59). *In vivo* in the U87 xenograft glioma model in athymic nude mice, the inhibition of mGluR1 also decreased tumor growth. However, the intraperitoneal administration of the inhibitor (BAY36-7620 and Riluzole) to mice could target other cells such as immunosuppressive immune cells in addition to U87 cells xenograft. Here, the inhibitory role of BAY36-7620 and Riluzole on the proliferation of U87 cells *in vivo* must be nuanced, since the role of the immune cells cannot be excluded. An interesting approach to exclude the potential involvement of immune cells in the inhibition of tumor growth by mGluR1 can be by the use of humanized HLA-matched mice. Humanized transgenic mice expressing several modified HLA class I molecules are used to mimic the human antigen presentation process which helps evaluate T-cell- based immune response in cancer (63). In this context, the evaluation of the difference in tumor growth between humanized HLA mice and athymic nude mice can provide a clear view of the role of immune cells in the tumor suppressor role of mGluR1 in glioma. The use of U87 mGluR1 knockout cells can also be used to study the specific role of mGluR1 for the *in vivo* study of this model.

##### 4.1.1.2 mGluR1 and breast cancer

In a cohort of 394 patients with breast cancer, mGluR1 was expressed in 56% (219) of the samples, whereas 46% (175) of the samples did not express mGluR1 (64). There was no correlation between mGluR1 and the overall survival of patients with breast cancer, but the expression of mGluR1 was positively correlated with the expression of the estrogen receptor (ER-positive) and progesterone receptor (PR-positive) in 60% of the sample (64). The expression of the hormone receptors (estrogen and progesterone) could increase the expression of mGluR1, contributing to the malignant behavior of breast cancer. This observation is in agreement with previous studies revealing that GluR1 might contribute to the malignant behavior of breast cancer and increase cell proliferation (60, 65), suggesting that this receptor alone is not sufficient to trigger breast cancer. This is consistent with the heterogeneous expression of mGluR1 in patients with breast cancer. Finally, the activation of mGluR1 in MDA-MB-231 breast cancer cells can release the cytokines and chemokines (CXCL1, IL6, IL8) that activate the immune-suppressive immune cell promoting the malignant transformation of breast cancer cells (66).

As reported in the previous paragraph, the involvement of mGluR1 in breast cancer, especially in the late stage (migration and invasion) was also shown by Banda et al. (65). The expression of mGluR1 was significantly increased in human breast cancer tissue and TNBC (triple negative breast cancer) cell line (BT549), whereas mGluR1 was not detected in normal tissue at the mRNA and protein levels. To investigate the expression of mGluR1 in the progression of breast cancer, mGluR1 was overexpressed in the MCF10 progression series of cell lines (premalignant members of the MCF10A series). Overexpression of mGluR1 did not have any effect on the proliferation, invasion, and colony formation in soft agar. However, the overexpression of mGluR1 in the MCF10AT1 cells (which represent atypical ductal hyperplasia) significantly increased the proliferation, migration, and invasion of cells (65). Inhibition of mGluR1 by the mGluR1 inhibitor BAY36-7620 decreased proliferation, migration, and invasion. The MCF10AT1 xenografts in nude mice followed by the overexpression mGluR1 developed multiple foci of invasive carcinoma in over 90% of lesions (65). These observations suggest that mGluR1 promotes migration and invasion in premalignant and malignant TNBC cells, but not in the nontransformed epithelium.

Angiogenesis has also been shown to be regulated by mGluR1 in breast cancer. Angiogenesis is characterized by the formation of new blood vessels, which play a crucial role in the migration, growth, and differentiation of endothelial cells during tumor progression. The expression of mGluR1 in four different types of endothelial cells was detected at different levels, with a higher expression in HUVEC and HMEC-1 compared to HDEC and HLEC, although the difference was not significant (67). The treatment of these four types of endothelial cells with BAY36-7620 and YM298198 (selective mGluR1 antagonists), as well as Riluzole, at various concentrations for up to 3 days revealed that HUVEC and HMEC-1 cells were more sensitive to inhibition by either Riluzole (85% and 70%, respectively) or BAY36-7620 (90% and 91%, respectively) at the highest concentration tested, which is consistent with the higher expression of mGluR1 in those cells. The treatment of HUVECs and HMEC-1 with Riluzole and BAY36-7620 at a high concentration inhibited the formation of tubes on Matrigel by Riluzole (48% and 60%, respectively) and BAY36-7620 (98% and 96%, respectively) in a dose-response manner. Interestingly, they found that BAY36-7620, which is a selective mGluR1 antagonist showed a greater inhibitory effect on tube formation than Riluzole in HUVECs cells. The *in vivo* treatment of the MDA-MB-231 xenograft model, with Riluzole used in a medical trial for breast cancer, inhibited tumor progression up to 50% in the 4T1 mice, as early as day 9 compared with the control, suggesting that mGluR1 can promote tumor growth through the regulation of angiogenesis (67). Moreover, the intraperitoneal injection of Riluzole or BAY36-7620 resulted in a significant reduction in microvessel density of up to 80% in xenograft proving the fundamental role played by mGluR1 in tumor growth (60). It has been shown that the activation of mGluR1 induces the release of vascular endothelial growth factor (VEGF) through the activation of different protein kinases including PKA, CaMKs, MAPKs, or PI3K (67), but this needs to be confirmed by further evidence.

##### 4.1.1.3 mGluR1 and melanoma

In the early 2000s, the progression of melanoma was studied by the use of mice mutant, TG3, which is predisposed to develop multiple melanomas affecting the pinnae of the ear, perianal region, eyelid, snout, trunk, and legs (68). mGluR1 was expressed in pinnae tumors but not in normal pinnae from C57BL/6J mice and in the mouse melanocyte cell line (melan-a). To further confirm the involvement of mGluR1 in melanocytic neoplasia, another transgenic line with mGluR1 expression regulated by a melanocyte-specific promoter, the dopachrome tautomerase was created. Similar to the original TG3, the Tg(mGluR1)EPv line was susceptible to developing melanoma, supporting the role of mGluR1 in the transformation of melanocytes (68). The expression of mGluR1 only in melanocytes of NIH3T3 and melan-a cells of transgenic Tg(mGluR1) induced the development of pigmented lesions on the pinnae and tail at 5-6 months of age, which progressed into raised lesions by 6-7 months (68). mGluR1 might directly influence the growth of melanocytes through the release of growth factors or changes in cell adhesion by adjacent keratinocytes. Interestingly, the development of melanoma was observed only in transgenic mice with the expression of mGluR1 in melanocytes, indicating that mGluR1 could be the main contributor to the development of melanoma in this model (68). One limitation of these transgenic mice is that there is hyperproliferation of melanocytes with limited transformation to fully malignant metastasis, which does not allow the investigation of advanced stages of melanoma. However, this model might be very useful to investigate the early stage of melanoma to find a potential therapy and limit the progression to the advanced stage. In the human sample, the expression of mGluR1 was not detected in normal melanocytes but mGluR1 was expressed in melanoma samples (68). This observation is in agreement with the fact that in a human model, mGluR1 might be mainly expressed in the advanced stage. The lack of the expression of mGluR1 in normal melanocytes could be attributed to the fact that the expression of mGluR1 can lead to the malignant transformation of melanocytes, thus requiring a tight regulation of mGluR1 by mechanisms that are not well understood.

The Neuron-Restrictive-Silencer-Factor (NRSF) can regulate several cell type-specific genes, and therefore may function as a negative regulator of tumorigenesis (69). H.J. Lee et al. (70) have reported the role of NRSF in the regulation of mGluR1. They showed that the alteration of the Neuron-Restrictive-Silencer-Factor (NRSF) and Neuron-Restrictive-Silencer-Element (NRSE) resulted in the aberrant expression of mGluR1 in primary human epidermal melanocytes (HEM). In normal physiological conditions, the expression of mGluR1 induces the activation of the canonical downstream signaling resulting in the activation of phospholipase C (PLC) and phosphoinositide (PI) (71, 72). Nevertheless, it was reported by Gelb et al. that the activation of mGluR1 by glutamate in SK2 and SK5 human melanoma cell lines resulted in an increase in cell viability at the concentration of 4.3 mM and 3.4 mM respectively for SK2 and SK5 independent of the canonical PLC and PI signaling pathways. They found that the activation and expression of mGluR1 induce the growth of human melanoma cells *in vitro* by an autocrine control of the availability of glutamate in the TME to sustain the growth of tumor cells, rather than the activation of canonical PLC and PI signaling (73). This was confirmed by the use of the natural agonist of mGluR1 (glutamate) which was correlated with an increase in cell viability in melanoma cells but failed to stimulate phosphoinositide (PI) hydrolysis, indicating that the activation of mGluR1 did not follow the canonical PLC/PI signaling in melanoma. In contrast, the synthetics mGluR1 agonist quisqualate or DHPG failed to stimulate phosphoinositide (PI) hydrolysis as well as increase cell viability (74). The caveat of the research is that glutamate act on its different receptors that might be expressed on the human melanoma cells used in the study, therefore, the mentioned observation is a net effect from different receptor activation. Thus, the true role of mGluR1 in human melanoma needs further investigation. The reason why the natural ligand of mGluR1 (glutamate) but not the synthetics agonist (quisqualate and DHPG) increased in cell viability is not well known, but the internalization of glutamate could be necessary for mGluR1 to induce effects on melanoma cells. Thus, the use of glutamate should be preferred over other synthetic agonists to investigate the role of mGluR1 in human melanoma (74).

##### 4.1.1.4 mGluR5 and glioma

This receptor has also been involved in increasing the viability and proliferation of glioma cell lines in hypoxic conditions. Four different types of human glioma cell lines (LNT-229, LNT-308, LNT-428, and G55) were treated with selective mGluR5 antagonist (MPEP), mGluR1-specific antagonist (CPCCOE), and group I mGluRs (mGluR1 and mGluR5) agonist DHPG as a negative control. In normoxia, the treatment of the four types of cells for 24 hours with the two antagonists (MPEP and CPCCOE) did not induce any effect on cell viability. However, under hypoxia, the treatment with the mGluR5 MPEP reduced the viability of glioma cells compared to the treatment with CPCCOE and increased the expression of mitochondrial respiratory function genes, such as PGC-1a and PGC-1b (75). Moreover, the treatment with DHPG, the group I agonist as a negative control, did not affect the viability and expression of the mitochondrial gene and failed to reverse the effects of the inhibition of mGluR5, showing that the effect involves the specific inhibition of mGluR5. Although this study revealed that the inhibition of mGluR5 in normoxia did not induce any change, another study found that the Riluzole treatment in normal conditions inhibited proliferation, induced apoptosis, and prevented migration of human osteosarcoma LM7 cells (76). The contradictory results could be attributed to the type of antagonist used in the two studies. While MPEP is a selective antagonist of mGluR5, Rulizole is a non-selective antagonist of mGluR5, acting on both mGluR1 and mGluR5. The authors found that LM7 cells express mGluR5, but they could not detect the expression of mGluR1. Nevertheless, the capacity of Rulizole to inhibit mGluR1 cannot be excluded and could explain the reason why the use of Rulizole decreases cell proliferation and migration in normoxia. In hypoxia, the increased expression of the mitochondrial gene might involve the phosphorylation of the AKT signaling pathway, rather than the activation of ERK that is activated in a traumatic neuronal injury through PKC (77). The evidence of the implication of AKT in mGluR5 mediates cancer cell proliferation is shown by the use of the AKT agonist SC79. Indeed, the co-treatment of glioma cells with SC79 (AKT activator) and MPEP (mGluR5 antagonist) reverses the effect of the mGluR5 inhibition in glioma cells, indicating that AKT as a downstream of mGluR5 can regulate cell viability in hypoxia and normal conditions (75, 76).

##### 4.1.1.5 mGluR5 and melanoma

As a member of group I mGluRs, mGluR5 was also reported to be involved in melanoma. As mentioned above, mGluR5 activates PLC, followed by the release of intracellular Ca^2+^, which subsequently activates the protein kinase C (PKC) (78). The binding of calmodulin (CaM) to mGluR5 enhances the expression of the receptor and the release of Ca^2+^ (79). In contrast, the PKC phosphorylation of serine 901 (S901) inhibits CaM binding and decreases surface expression. As PKC competes with CaM, the mutation of S901 in the mGluR5 blocks the phosphorylation of PKC and increases the expression of mGluR5 by the binding of CaM (80). Thus, the generation of transgenic mice with the S901 mutation in mGluR5 in the Thy1 promoter region can be used to investigate the role of the receptor in melanoma. Compared to mGluR5 wild-type, Thy1-mGluR5 S901A mutant mice developed melanoma. The use of the transgenic lines in which the expression of mGluR5 is specifically targeted to melanocytes (TRP1 promoter), resulted in the development of severe melanoma on ears, nose, and tail. Transgenic mice are characterized by the hyperpigmentation of the ear and tail in the early stage followed by melanoma tumor formation in the pinnae and tail at a later stage. Additionally, the transgenic mice showed a very aggressive melanoma with the presence of melanoma cells in muscle and bone. The spread of melanoma in other organs is a hallmark of the aggressiveness of melanoma, indicating that the expression of mGluR5 increases the aggressiveness of melanoma (80). As mentioned previously on the role of mGluR1 in melanoma, mGluR5 might be mostly involved in the progression of melanoma, as indicated by the lack of the expression of mGluR5 in the normal skin of the C57BL/6J, while the expression of mGluR5 was very high in the tumor sample. The high expression of mGluR5 in human tumor tissue compared to normal tissue where there was a lack of expression of mGluR5 agrees with the transgenic mice data. There was a significant increase in the phosphorylation state of ERK in transgene-positive mice compared with transgene-negative mice, suggesting that ERK is implicated in the proliferation of melanoma cells (80). mGluR5 and mGluR1 belongs to the group I mGluRs. The possible cooperation of these two receptors in melanomagenesis was investigated by the generation of mice that were null for mGluR5 and carried the TG-3 mutation, which is characterized by the ectopic expression of mGluR1 in melanoma. mGluR5 homozygous and transgenic mice develop pigmented lesions that greatly resemble melanoma tumors in TG-3 mice, suggesting that mGluR5 is not required for the oncogenic role of mGluR1 in melanocytes (81).

##### 4.1.1.6 mGluR5 and oral squamous cell carcinoma (SCC)

In oral squamous cell carcinoma (SCC), the expression of mGluR5 is associated with a poor outcome (62). The oral SCC tissue showed feeble expression of mGluR5 in the basal layer of the normal oral squamous cell epithelium, and out of 131 oral SCC samples, only 42 (32%) exhibited a strong positive expression of mGluR5. Moreover, the expression of mGluR5 was positively correlated with the stage of oral SCC, with a low or no expression in the early stage, and a relatively strong expression in the advanced stage, showing the correlation between mGluR5 and the progression of oral SCC. The *In vitro* assay demonstrated that the agonist of mGluR5 DHPG even at a high concentration (100 μM) did not induce a change in the viability or cell proliferation, but enhanced the migration and invasiveness of the HSC3 oral cancer cells. Besides, the inhibition of mGluR5 by the selective antagonist MPEP inhibited the abovementioned effects (62). These results are consistent with the possible role played by mGluR5 in the late stages of progression, but not in the onset of oral SCC, given the fact that the expression of mGluR5 increases in the late stage of oral SCC.

In neuronal and non-neuronal cancer, the activation group 1 mGluRs (mGluR1 and 5) seems to be tumor-promoting, thus the strategies that inhibit the expression of group 1 mGluRs can be important for treating different types of cancer in which group 1 mGluRs is involved. Furthermore, the weak or the absence of the expression of mGluR1 and mGluR5 in normal cells compared with cancer cells allow making the hypothesis that these receptors are not involved in the onset of cancer, rather, the activation of mGluR1 and mGluR5 might occur in the cancer cell or in a cell undergoing a malignant transformation which can contribute to the progression of cancer and the malignant behavior cancer cells. However, the roles of mGluR1 and mGluR5 in other tumors and the specific mechanism remain to be investigated. In addition, it is still unknown whether the roles of mGluR1 and mGluR5 in tumor progression are compensative or redundant.

#### 4.1.2 Group II mGluRs and cancer

The expression of mGluR2 and 3 have been investigated in neuronal cells, such as U87MG human glioma cells (82) and non-neuronal mouse B16F10 melanoma cell lines (83).

##### 4.1.2.1 mGluR2/3 and glioma

In glioma, which is one of the most aggressive brain cancers, the mechanisms of group II mGluRs involved in the regulation of tumor growth have been studied several years ago. In addition to the role played by the mGluR1 in the activation of PI3K/AKT/mTOR in glioma (59), MAPK and PI3K pathways are activated by mGluR2/3 in glioma (82). These signaling pathways play a very important role in cell proliferation. Indeed, subcutaneous tumor growth of human glioma U87MG cells in nude mice was significantly suppressed by LY341495, a mGluR2/3 antagonist. *In vitro*, LY341495 treatment inhibited activation of MAPK and PI3K pathways in U87MG cells, indicating the regulation of these signaling pathways by mGluR2/3 in glioma (82). Whether the use of the synthetic agonist can reverse the tumor suppression effects of the mGluR2/3 antagonist mentioned above is not well established, but it might be possible that the use of the natural ligand of mGluR2/3 and the use of the synthetic agonist can stimulate tumor cell proliferation and tumor growth *in vivo*.

##### 4.1.2.2 mGluR2/3 and melanoma

**Figure 3:**
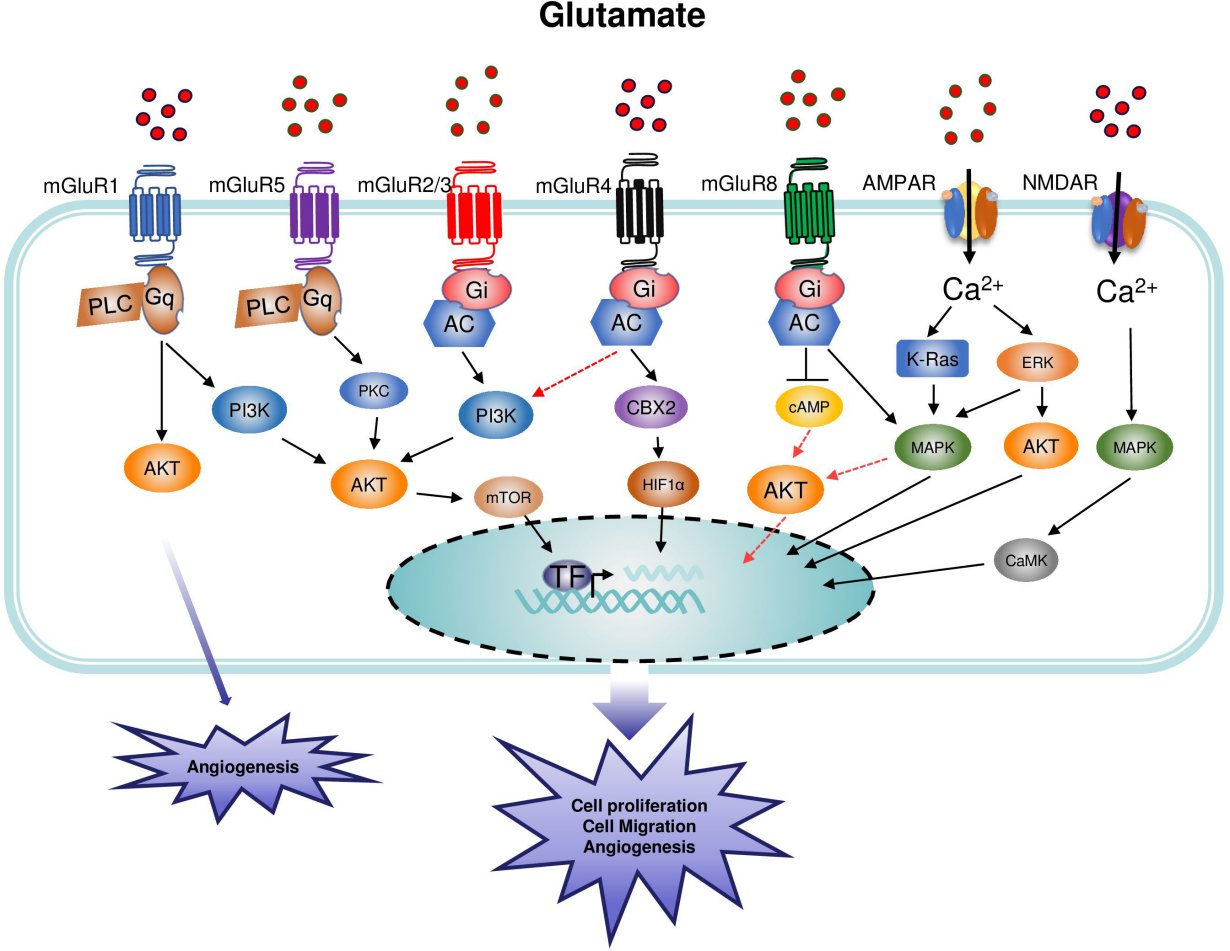
Main signaling pathways involved in the regulation of cancer by metabotropic glutamate receptor (mGluR) and inotropic glutamate receptor (iGluR). Group I mGluRs are coupled to phospholipase C (PLC) while groups II and III are coupled to adenylate cyclase (AC). NMDAR are ligand-gated ion channels that mediate a rapid depolarization of the membrane. The binding of glutamate to the glutamate binding site acts as a first messenger and induces the depolarization of the membrane leading to the influx of Ca^2+^ in the intracellular space, followed by the activation of the corresponding signaling pathways. Glutamate is the agonist of AMPAR, and the channel opens when two sites are occupied by glutamate. Within the receptor, the GluN2 subunit plays a critical role in the determination of the Ca^2+^ permeability of the AMPAR. Upon binding of the amino-acid glutamate to its receptors, the intracellular signal transduction mechanisms induce the activation of different signaling pathways leading to cell proliferation and tumor growth.

The role of mGluR2/3 in melanoma has been studied (83). In the B16F10 melanoma subcutaneous tumor model, the treatment by the intraperitoneal injection of LY341495 inhibited melanoma growth in C57BL/6J wide-type mice. In this study, the authors did not detect the expression of mGluR2/3 on tumor cells and the direct effect of LY341495 on tumor cells. Although mGluR2/3 expression on myeloid-derived suppressor cells (MDSCs) and the change of tumor-infiltrating immune cells after LY341495 treatment were evaluated, it remains unclear whether these effects are caused by the direct effect of LY341495 on a tumor cell or MDSCs. The role of mGluR2/3 in MDSCs will be discussed below in the “the role of glutamate receptors in immune cells” section. So far, the studies on the roles of mGluR2/3 have been based on the use of agonists and antagonists that target both receptors, therefore specific silence/overexpression of mGluR2 and mGluR3 will be necessary to interpret their independent roles in tumors (**Figure 3**).

#### 4.1.3 Group III mGluRs and cancer

This group has been implicated in various cancer. The expression of group III mGluRs was detected in several types of cancers, including but not limited to colorectal, laryngeal squamous cell, breast cancers, osteosarcoma, and melanoma (25). To date, very few studies have investigated the involvement of mGluR6 and 7 in cancer, further studies on the role of mGluR6 and 7 in cancer are needed.

##### 4.1.3.1 mGluR4 and glioblastoma

Different from the report that the activation of mGluR1 and mGluR5 is associated with an increase in tumor cell proliferation and migration as well as a poor outcome in glioma (59, 75), the activation of mGluR4 is associated with a decrease in cell proliferation and the reduction of cell viability in glioblastoma multiforme (GBM) in a time and dose-dependent manner after 24, 48, and 72-hours treatment with 30 and 50 μM of mGluR4 specific agonist VU0155041 (84). The mGluR4 is highly expressed by neural stem/progenitor cells and embryonic stem cells (84). This receptor is also expressed by the LN229 glioblastoma cell line. The pharmacological activation of mGluR4 by the specific agonist VU0155041 induces apoptosis and reduces the viability as well as the proliferation of LN229 cells (84). In this study, the authors found that the activation of mGluR4 was negatively correlated with the Glioma-associated oncogene homolog 1 (Gli-1), a transcription factor that regulates cell proliferation and differentiation during CNS development. Furthermore, silencing mGluR4 could reverse the inhibitory effects of VU0155041 on Gli-1 expression, suggesting that the activation of mGluR4 can suppress the multiplication of glioblastoma cells *via* Gli-1 (84).

##### 4.1.3.2 mGluR4 and colorectal cancer

Several years ago, the expression of mGluR4 was detected in colon adenocarcinoma (85). A high level of mGluR4 was correlated with a poor prognosis of colorectal cancer. The analysis of 241 colorectal cancer patients showed that the expression of mGluR4 was altered in 122 patients (51%). Among those 122 patients, 110 patients (46%) showed a loss of mGluR4 expression, and 12 patients (5%) had an overexpression of mGluR4. The analysis of some clinicopathologic parameters of patients with colorectal cancer, including differentiation type (poorly differentiated or well-differentiated), tumor size, location, lymphatic or venous invasion, lymph node or distant metastasis, stage, microsatellite instability, or p53 expression and mGluR4 expression indicates a correlation only with the differentiation type. Indeed, there was a loss of mGluR4 in moderately to poorly differentiated types, compared to well-differentiated types, besides, there was no correlation between colorectal cancer and the other clinic-pathologic parameters. *In vitro*, the treatment of different colorectal cancer cell lines (SNU-61, SNU-81, SNU-407, SNU-1033, SNU-1047, SNU-C2A, SNU-C4, and SNU-C5) with the mGluR4 agonist (L-AP 4) increased the proliferation only in SNU-81 cells. All these results are consistent with the hypothesis that mGluR4 could control only some parameters involved in the pathogenesis of colorectal cancer. It was speculated that the mGluR4 is necessary, but not sufficient alone to induce colon adenocarcinoma. Different signaling pathways of which the most common AKT/PI3K are associated with the activation of mGluRs in cancer, however, the signaling linking mGluR4 in colon adenocarcinoma is not yet well established (85).

##### 4.1.3.3 mGluR4 and osteosarcoma

In 2004, the expression of mGluR4 was detected in MG-63 osteoblast-like osteosarcoma cells (86). Following this observation, Yang et al. investigated the relationship between the expression of mGluR4 and the prognosis of osteosarcoma patients. The study demonstrated that the expression of mGluR4 was significantly increased in osteosarcoma tissues compared with normal tissues at the mRNA and protein levels. Also, the expression was correlated with the Enneking stage and metastasis of osteosarcoma, suggesting that mGluR4 is implicated in the onset and development of osteosarcoma (87). In this study, the antibody clone was not stated and thus, it is unclear whether the specificity of the antibody is validated by other studies. Thus, further studies are needed to confirm the findings of this work. However, a recent study showed that there was not a significant difference in the expression of mGluR4 between osteosarcoma tissue and normal tissue. The same observation was performed between osteosarcoma cells (Saos-2) and human osteoblasts hFOB1.19, suggesting that mGluR4 might play a role only in the progression of osteosarcoma. This hypothesis is in agreement with the *in vitro* results, showing that physiological activation of mGluR4 (by glutamate at the concentration of 3-20 µM) (78) did not change the proliferation and migration of cells. However, the overexpression of mGluR4 decreased cell proliferation, migration, and invasion of human osteosarcoma cells (MG-63, U2OS, Saos-2). The low expression of mGluR4 in osteosarcoma cells could explain the reason why the physiological activation of mGluR4 did not induce a change in the proliferation while the overexpression decreased cell proliferation, migration, and invasion. The authors of this study suggested that the signaling pathway linking mGluR4 and osteosarcoma might involve the mGluR4/CBX4/HIF-1α signaling pathway (**Figure 3**) (88). Of note, a positive allosteric VU0364439 of mGluR4 did not affect the proliferation, migration, and invasion of osteosarcoma cells (86, 88). There is still a question about the reason for the contradictory results on the expression of mGluR4 in normal and osteosarcoma tissue between the two studies. A possible explanation can be attributed to the lack of heterogeneity and the limited sample size of tumors. However, further investigations are needed to clarify the role of mGluR4 in osteosarcoma.

##### 4.1.3.4 mGluR8 and neuroblastoma

mGluR8 plays a dual role, either a protective or neurotoxic role in neuroblastoma cells according to the type of cells (undifferentiated or differentiated cells) (89). The co-treatment of undifferentiated (UN) and retinoic acid differentiated (RA) SH-SY5Y neuroblastoma cells with a selective allosteric modulator (PAM: AZ12216052) and orthosteric agonist ((S)-3,4-DCPG) combined with chemotherapeutic drugs, irinotecan and cisplatin showed interesting results. The treatment of neuroblastoma cells with AZ12216052 resulted in the resistance to cytotoxic effects of irinotecan or cisplatin, whereas the use of (S)-3,4-DCPG induced partial resistance of UN- and RA-SH-SY5Y cells only to the cisplatin treatment (89). In differentiated (RA-) SH-SY5Y cells, the use of the two mGluR8 agonists significantly enhanced the toxic effects of doxorubicin and irinotecan against RA-SH-SY5Y cells. These results suggest that, in undifferentiated cells, the activation of mGluR8 is tumor-promoting whereas mGluR8 activators can improve the sensitivity of differentiated cells, such as non-malignant cells, to chemotherapy drugs (89). The tumor suppressor activity of mGluR8 in glioma and glioblastoma was also shown by the overexpression and the downregulation of mGluR8 compared with native cells (90). The exogenous overexpression of mGluR8 in human glioblastoma U87-MG and LN18 decreased the proliferation. Silence of mGluR8 in neuroblastoma SH-SY5Y was followed by increased proliferation. As one of the most aggressive tumors, the therapeutic approach for the treatment of glioblastoma is still not satisfactory. The use of doxorubicin, irinotecan, or cisplatin has beneficial effects on different types of cancer, such as metastatic gastric cancer (91). The normal U87-MG cells were shown to be resistant to a different type of chemotherapeutic agent (doxorubicin, irinotecan, or cisplatin) even at high concentrations. In contrast, the increased expression of mGluR8 was correlated with the induction of apoptosis as well as the increase in the sensitivity to the chemotherapeutic agent (90). Another important aspect of this study is that the downregulation of mGluR8 in neuroblastoma SH-SY5Y could be important to study the mechanism of chemoresistance to the treatment with a chemotherapeutic agent.

##### 4.1.3.5 mGluR8 and squamous cell lung carcinoma (LUSC)

Contrary to the tumor suppressive activity of mGluR8 in the neuronal tumor, the activation of this receptor in non-neuronal tumors is correlated with a poor prognosis. The activation of mGluR8 in squamous cell lung carcinoma (LUSC) promoted the proliferation and survival of LUSC tumor cells through the inhibition of cAMP and the activation of MAPK (**Figure 3**) (92).

In lung cancers, a high level of intracellular glutamate is correlated with the expression of the cystine/glutamate antiporter (xCT/SLC7A11) (93, 94). The crucial role played by the xCT system in the maintenance of the concentration gradients of glutamate and cystine across the plasma membrane is important for the synthesis of glutathione (GSH), which is the most abundant antioxidant within all cells (18). The expression of xCT in lung cancer is correlated with the export of glutamate from the intracellular milieu followed by the import of cystine for the synthesis of GSH. This process induces the accumulation of glutamate in the extracellular compartment (93). GluRs are expressed on the cell surface, therefore, the accumulation of extracellular glutamate could induce the activation of surrounding cancer and immune cells increasing cell proliferation or anti-tumor response. However, there are few pieces of evidence on the role of glutamate exported by the xCT system in the activation of GluRs expressed on the surface of cancer or immune cells, therefore further investigation to highlight the relationship between the glutamate transporters and GluRs will be important to understand the role of glutamate in some cancer cells.

##### 4.1.3.6 mGluR8 and breast cancer

The tumor-promoting role played by mGluR8 in non-neuronal tumors was observed in the work of Zhang et al. in breast microRNA cancer (95). They found that the mRNA level and the protein expression of mGluR8 were significantly higher in breast cancer cell lines including HCC1937, Bcap-37, MDA-MB-231, MCF7, and SK-BR-3 compared with that of the normal breast cell line Hs 578Bst. The same observation was made in breast cancer tissues compared with normal tissues. Moreover, the increased expression of mGluR8 in cancer tissues and cells was positively correlated with the poor prognosis and the shorter overall survival of patients with breast cancer. The proliferation, migration, and invasion of cancer cells were repressed by the use of microRNA targeting mGluR8. The upregulation of miR-33a-5p in particular was reported to be inversely proportional to the proliferation and migration of triple-negative breast cancer (TNBC) cells. In breast cancer, the expression of miR-33a-5p was significantly decreased compared to the normal tissue, and the decreased expression of miR-33a-5p was correlated with poor survival of a patient with breast cancer. The miR-33a-5p suppressed the proliferation, migration, and invasion, acting as a tumor suppressor in breast cancer. Moreover, miR-33a-5p could downregulate mGluR8, which was verified by luciferase gene reporter assay (95).

In conclusion of the role of mGluR8 in cancer, we can highlight that the role played by mGluR8 in neuronal cancer seems to be opposite to the role played in non-neuronal cancer. Neuronal cancer cells release neurotrophic factors and cytokines, that can activate non-neuronal cancer cells leading to their proliferation (96). Glutamate is a crucial neurotransmitter in the brain; thus, the decreased expression could be harmful, therefore the activation of mGluR8 in the neuronal tumor can be a benefit in decreasing the tumor growth. In contrast, glutamate in non-neuronal cancer cells acts as a growth factor, and the activation of mGluR8 can promote tumor growth

### 4.2 Involvement of the inotropic glutamate receptor (iGluR) in cancer

#### 4.2.1 Association between NMDAR and cancer

The involvement of NMDAR in neuronal disorders and cancer is increasing due to the high expression of this receptor in neuronal and non-neuronal cells.

##### 4.2.1.1 NMDAR and melanoma

In melanoma, the expression of GRIN2A (Glutamate Ionotropic Receptor NMDA Type Subunit 2A) was reported to be mutated in 33% of melanoma samples (97). A similar study found that the mutation of GRIN2A was detected in 25% of melanoma samples. The activation of NMDAR by glutamate induces the influx of ca^2+^ activating the pro-apoptotic signaling such as p38 MAPK, followed by reduced cell proliferation, migration, and invasion (98). Mutation of GRIN2A in NMDAR decreased the activation of p38 MAPK. In addition, the overexpression of the mutant GRIN2A in 31T and SK-Mel-2 melanoma cell lines increased the migration compared to both wild-type GRIN2A, whereas the use of a specific NMDAR2A (GRIN2A) antagonist, TCN-213 reversed the effect mentioned. Additionally, the knockdown of the GRIN2A in the GRIN2A mutant caused a slight reduction in proliferation compared to vector control cells, while the knockdown of wild-type GRIN2A resulted in increased proliferation of cells (98). This indicates that the GRIN2A mutation acts as a proto-tumor in melanoma, given the fact that the mutation of GRIN2A in melanoma cells increases the proliferation of melanoma cells (98). Another study has investigated the role of NMDAR in melanoma using a selective antagonist memantine approved by the FDA for Alzheimer’s disease (99, 100). The incubation of mouse melanoma K1735-M2 cells for 96 hours with both MK-801 and memantine decreased cell proliferation at the concentrations of 500 mM and 300 mM for MK-801 and memantine, respectively. In contrast, the treatment of cells with 500 mM of APV or NBQX, a non-selective NMDAR antagonist, did not reduce the cell proliferation even after 96 hours of incubation, indicating that the decrease in the cell proliferation involved NMDAR, but not AMPAR and KA receptors, since APV or NBQX can activate NMDAR, AMPAR and KA receptors (101). Indeed MK-801 and memantine may exert their effect in a signaling pathway different than the one used by APV. Memantine can act through the 5-hydroxytryptamine receptor 3, the α7 and/or α4β2 nicotinic receptors, and the dopamine receptors, while MK-801 might act on the α7 and α4β2 nicotinic receptors (101, 102). However, the results of the study couldn’t confirm whether the use of MK-801 or memantine activates the above signaling in melanoma or not.

To further investigate the role of NMDAR antagonist in the combined therapy with different antiestrogen tamoxifen (TAM), 4-hydroxytamoxifen (OHTAM), or endoxifen (EDX), MK801 was selected (101), since memantine can affect mitochondrial function (103). The combination of the NMDAR antagonist MK-801 (100 mM) with different antiestrogens (TAM, OHTAM, and EDX) at a concentration of 5 mM, strongly reduces melanoma cell proliferation and viability, even more than the observation made when the cells were treated only with MK-801 (500 mM). Also, the treatment of cells with MK801 alone (100 mM) did not induce a change in cell proliferation and viability. All these observations indicate that the treatment of cells with antiestrogen therapy enhances the efficacity of NMDAR antagonists. Although the signaling pathway linking the antiestrogens with MK801 is not known, the combination of the two therapy may induce the activation of both MAPK and extracellular signal-regulated kinase (ERK1/2), since the MK801 has been shown to reduce the proliferation of cancer cells through the inhibition of ERK 1/2 (104), it’s, therefore, reasonable to think that this signaling is involved in the reduction of melanoma cells growth after NMDAR antagonist treatment.

##### 4.2.1.2 NMDAR and breast cancer

In triple-negative breast cancer (TNBC), the role of microRNA (miRNA) in the regulation of NMDAR has been reported. The transfection of MDA-MB-231 cells with miR-129-1-3p suppressed tumor growth, and reduced cell proliferation (105). The increase in Ca^2+^ is considered to be central in the progression of breast cancer, and the miR-129-1-3p transfection of MDA-MB-231cells was followed by the decrease in intracellular Ca^2+^ and the activation of GRIN2D (Glutamate Ionotropic Receptor NMDA Type Subunit 2D), indicating that the miR-129-1-3p can suppress the development of TNBC by the regulation of MNDAR (105). However, the role of miR-129-1-3p mentioned in this study must be confirmed by further studies. For example, the use of an NMDAR antagonist can be used to confirm the involvement of miR-129-1-3p in the regulation of NMDAR and the inhibition of tumor growth. Moreover, the expression of other iGluRs such as AMPAR and KA can be detected to exclude their effect in the inhibitory effect of miR-129-1-3p. Although miRNAs can regulate different aspects of cancer biology, including angiogenesis, drug resistance, apoptosis, proliferation, invasion, and metastasis (105), acting as tumor suppressors in several types of cancer a lot needs to be done to link the beneficial effect of miRNA with iGluRs in different types of cancer.

##### 4.2.1.3 NMDAR and pancreatic neuroendocrine tumorigenesis (PNET)

The signaling pathways related to NMDAR have been well studied in a mouse model of pancreatic neuroendocrine tumorigenesis (PNET) and selected human cancers (106). The expression of NMDAR was increased in the βTC-3 cancer cell line and PNET tumor tissue, particularly the invasive fronts compared to normal cells and healthy patients. Also, the expression of NMDAR was correlated with a poor prognosis of patients with PNET. The *in vitro* treatment of βTC-3 cancer cells with MK-801 time-dependently decreased the proliferation and increase apoptosis. Moreover, the use of MK-801 blocked the invasiveness of βTC-3 cancer cells assessed by the flow-based invasion assay, while the use of the AMPAR antagonist GYKI5246 had a modest anti-invasive activity. *In vivo*, the use of MK-801 was sufficient to inhibit cell proliferation at a level comparable to that observed *in vitro*, however, although the use of MK-801 *in vivo* could reduce the invasion of cells, the reduction was not as striking as *in vitro*. The reason could be related to the fact that MK-801 has a very short half-life, of about an hour, therefore short-term treatment could reduce the efficacity of tumor invasion (106). As mentioned above, the beneficial effects of MK-801 and memantine in the inhibition of the progression of cancer depend on the stage of cancer.

Another study on Pancreatic cancer revealed that the expression of NMDAR was not detected in normal human pancreas, ovary, kidney, breast, and lung tissues. Protein expression of GluN1 or GluN2B was also not detected in the mouse pancreas, ovary, kidney, lung, liver, heart, intestine, and skeletal muscle, but both were present in extracts from the brain. In contrast, GluN1 and GluN2B were highly expressed by PanC-1, HPAC-1, and BXCPC-3 cells (107). The treatment of the cells with MK801 for 48 hours at a concentration of 200 μM significantly reduced the cell viability compared to the control. The *in vivo* treatment of mice with MK801(0.3 mg/kg body weight) inhibited the tumor growth of PanC-1 tumor xenografts in nu/nu mice. More interestingly, the treatment of mice with the GluN2B antagonist ifenprodil (2.5 mg/kg body weight) prevented the growth of PanC-1 tumor xenografts and decreased the tumor size by almost half, indicating that ifenprodil is more effective in the inhibition of NMDAR *in vivo* (107). This can explain why ifenprodil has been approved for clinical use in Japan and France, and a similar compound in the USA (107). The activation of NMDAR can activate different types of signaling pathways. Thus, the activation of the MEK-MAPK pathway by NMDAR has preferentially induced the proliferation and survival of cancer cells, whereas the activation of the CaMK pathway, in particular CaMK–IV, plays a major role in invasion (106). Consistent with all this, the authors of the study found that the *in vitro* treatment of βTC-3 cancer cells with MK-801 induced the activation of both MEK-MAPK and CaMK signaling pathways, probably because in cell conditions, the proliferation, anti-apoptotic, and invasion occur within a relatively short period (72 hours) allowing the activation of proliferation and invasion at the same time (**Figure 3**). Although the mechanism of MK801 in PanC-1, HPAC-1, and BXCPC-3 cells was not investigated, we speculate that the above signaling pathways could be activated.

The available data indicate that the expression of NMDAR is increased in tumor tissues and cancer cells, supporting the pro-tumor role of this receptor in cancer development. Although the use of different inhibitors is effective in decreasing cancer cell proliferation, migration, and invasion, several elements are important to improve therapeutic strategies involving NMDAR and cancer. One concern to consider is the heterogeneous expression of the NMDAR in different types of cancer. Therefore, the inhibitory effects of NMDAR antagonists depend on the expression of NMDAR in those tumor cells. Thus, NMDAR antagonists may be more effective in patients with broader and higher expression/activation of the NMDAR signaling axis. Moreover, the use of combined therapy with conventional drugs targeting the tumor core along with NMDAR inhibitors targeting the invasive periphery can have more beneficial effects on the invasion of tumor cells (106).

#### 4.2.2 AMPAR and its role in cancer

The expression of AMPAR has been studied mostly in neuronal-associated cancer such as gliomas (38) and neuroblastoma, but also in non-neuronal cancers including pancreatic cancer and medullary thyroid cancer.

##### 4.2.2.1 AMPAR and glioma

**Figure 4:**
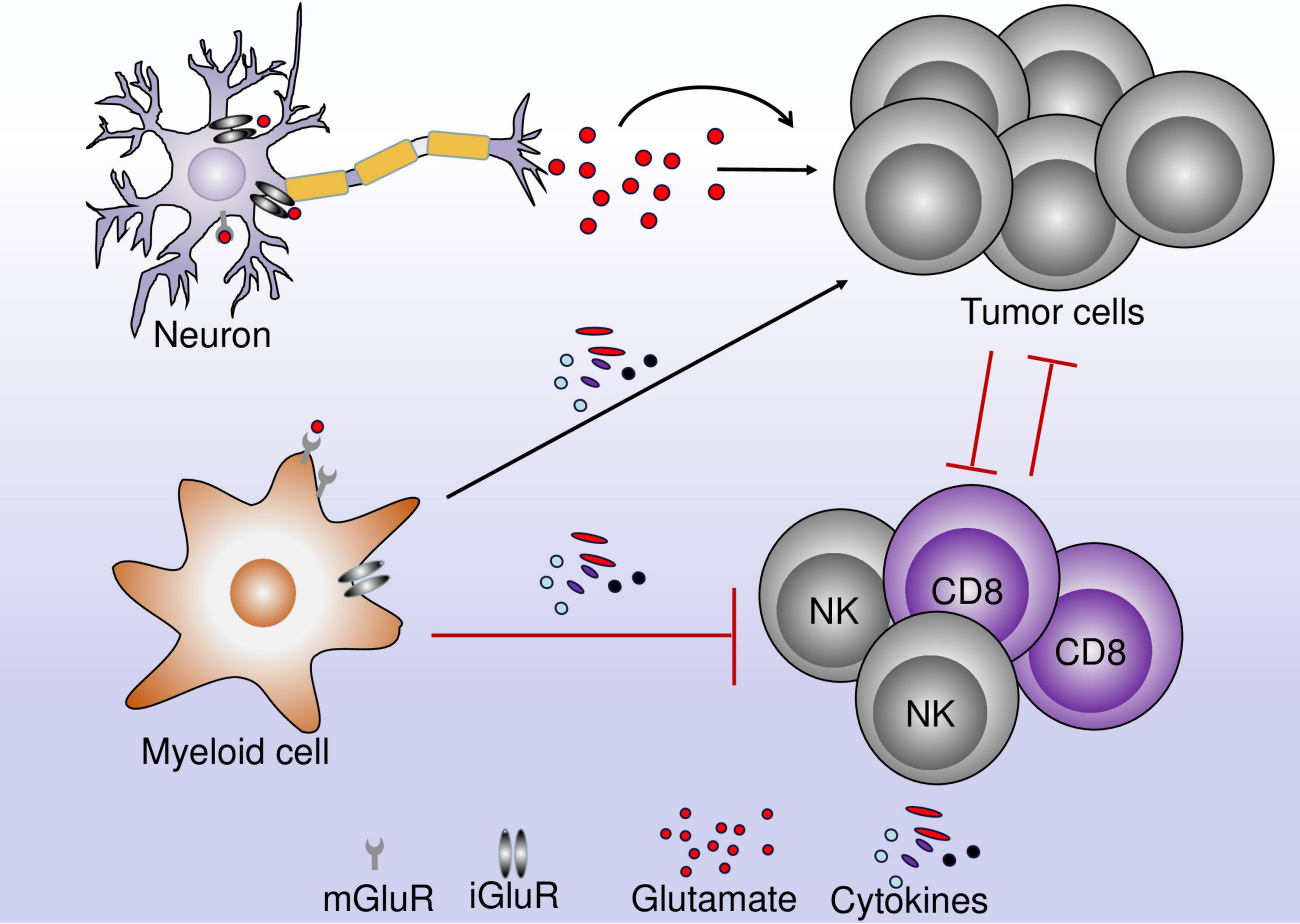
The role of glutamate in the interaction between neurons, immune cells, and tumor cells. A neuron can release neurotrophic factors and cytokines leading to the regulation of TME. These cytokines can act directly on tumor cells leading to their proliferation and tumor growth. Within the TME, immune cells and tumor cells interact together. Glutamate can bind to glutamate receptors on myeloid and lymphoid cells leading to an increase in the antitumor immune response or the inhibition of the antitumor immune response.

Photodynamic therapy (PDT) has been used clinically in the management of several types of tumors located in the lung, ovary, and brain. The role of AMPAR in PDT has been investigated several years ago (38). Treatment of glioma cells with PDT significantly increases the expression of AMPAR. Thus, the treatment of glioma cells with PDT increased the extracellular concentration of glutamate (three-time higher than the normal group) leading to an increase in Ca^2+^ influx (38). Glutamate released by glioma cells in the extracellular milieu caused excitotoxicity to the surrounding neurons by Ca^2+^ influx. This process allows the survival of glioma cells, while the normal surrounding neural cells undergo excitotoxic cell death (**Figure 4**) (108). During this process, the activation of immunosuppressive myeloid cells induces the release of cytokines that will subsequently promote tumor growth and inhibit effector immune cells such as T and NK cells. The inhibition of AMPAR using the non-competitive antagonist CNQX decreased the tumor cell-cytotoxic effects caused by the increase in Ca^2+^ influx indicating the protective role played by AMPAR inhibition in this process (38). The increase in Ca^2+^ influx is mediated by the calcium-permeable AMPAR-type glutamate receptors assembled from the GluR1 and/or GluR4 subunits (109). It is important to consider the subunit in the biology of AMPAR because, in contrast to GluR1 and GluR4 which are calcium-permeable, the GluR2 subunit is calcium-impermeable AMPA receptors. In glioblastoma, the aggressive growth of glioma cells was also correlated with the increase in Ca^2+^ influx and the activation of AMPAR, whereas the inhibition of AMPAR by the antagonist suppressed the growth of glioma cells and decreased the invasiveness of the tumor, indicating the direct involvement of AMPAR in the increased in tumor growth (109). The cooperation between the calcium-permeable AMPAR-type glutamate receptors and ERK–MAPK is necessary for the AMPAR to induce the growth of tumor cells (**Figure 3**). Thus, according to Poddar et al., 2017 (110), the expression of GluR2 can be decreased by the phosphorylation of ERK–MAPK. Moreover, the inhibition of ERK is associated with the decrease of the expression of GluR1 and GluR4 subunits while increased that of GluR2 in AMPAR. The previous study has also revealed, that in high-grade glioma cells, aggressiveness is correlated with the significant phosphorylation of ERK compared to low-grade gliomas, indicating that ERK is involved in the aggressive phenotype of glioma cells (111). Moreover, the inhibition of ERK-MAPK resulted in the reduction of the aggressiveness of glioma cells (**Figure 3**) (109).

In glioma, the increased surface expression of β1 integrin increases the invasion of the cell. The upstream signaling involved in this process includes the activation of GluR1. The increased expression of GluR1 is followed by the activation of ECM leading to cell invasion by the cooperation with β1 integrin (112).

##### 4.2.2.2 AMPAR and neuroblastoma

The activation of AKT serine/threonine kinase (AKT) in cooperation with ERK also governs the proliferation of cells in several types of cancer. Like other types of cancer, the activation of AKT has been associated with a poor prognosis of neuroblastoma. Nozawa et al. (113) have shown that the activation of AMPAR in glioblastoma was correlated with the increased phosphorylation of AKT and ERK, increasing the proliferation of KP-N-SI9s human neuroblastoma cell line. Furthermore, the treatment of KP-N-SI9s with Perampanel (200 μM for 48 hours), a non-competitive AMPAR antagonist that is used to control epileptic seizures, suppressed the proliferation of glioblastoma cells, *via* inhibiting the AKT/ERK signaling (**Figure 3**). The tumor-suppressing effect of Perampanel was mediated by inhibiting the proliferation of tumor cells rather than the induction of apoptosis (113). The apoptosis in glioblastoma might require a signaling pathway other than the one used by Perampanel (AKT/ERK signaling). The use of YM872, a competitive antagonist of AMPAR, to induce the apoptosis of glioblastoma cells, is in agreement with this assumption, confirming that perampanel suppresses tumor growth by inhibiting cell proliferation in AKT/ERK signaling (114).

##### 4.2.2.3 AMPAR and pancreatic ductal adenocarcinoma (PDAC)

In non-neuronal cancer, the activation of AMPAR in pancreatic ductal adenocarcinoma (PDAC) has been reported to be involved in the aggressive and invasive pancreatic cancer phenotype (115). The activation of AMPAR, particularly GluR1 and GluR2 subunits, induced a switch to invasive and migratory phenotype, *via* activation of the K-ras/MAPK cascade (**Figure 3**). Moreover, this phenomenon seems to be independent of Ca^2+^ influx since the inhibition of calcium-permeable GluR1 and calcium-impermeable GluR2 decreased the migration and invasion of cells, without having any impact on the proliferation of cells (115).

In the neuronal tumor, the increased concentration of glutamate can promote the survival of cancer cells by Ca^2+^ influx. As the permeability of the membrane to Ca^2+^ is determined by the GluR2 subunit of AMPAR, the inhibition of AMPAR could be beneficial in decreasing the growth of tumors in which AMPAR is highly expressed. However, it’s possible that in non-neuronal tumors, the activation of AMPAR promotes tumor growth by a mechanism independent of Ca^2+^ influx, therefore, further investigations on the role of AMPAR in the non-neuronal tumors are required to have a better understanding of the mechanism.

## 5 The role of glutamate receptors in immune cells

### 5.1 The metabotropic glutamate receptors and the regulation of the immune cells in TME

**Figure 5:**
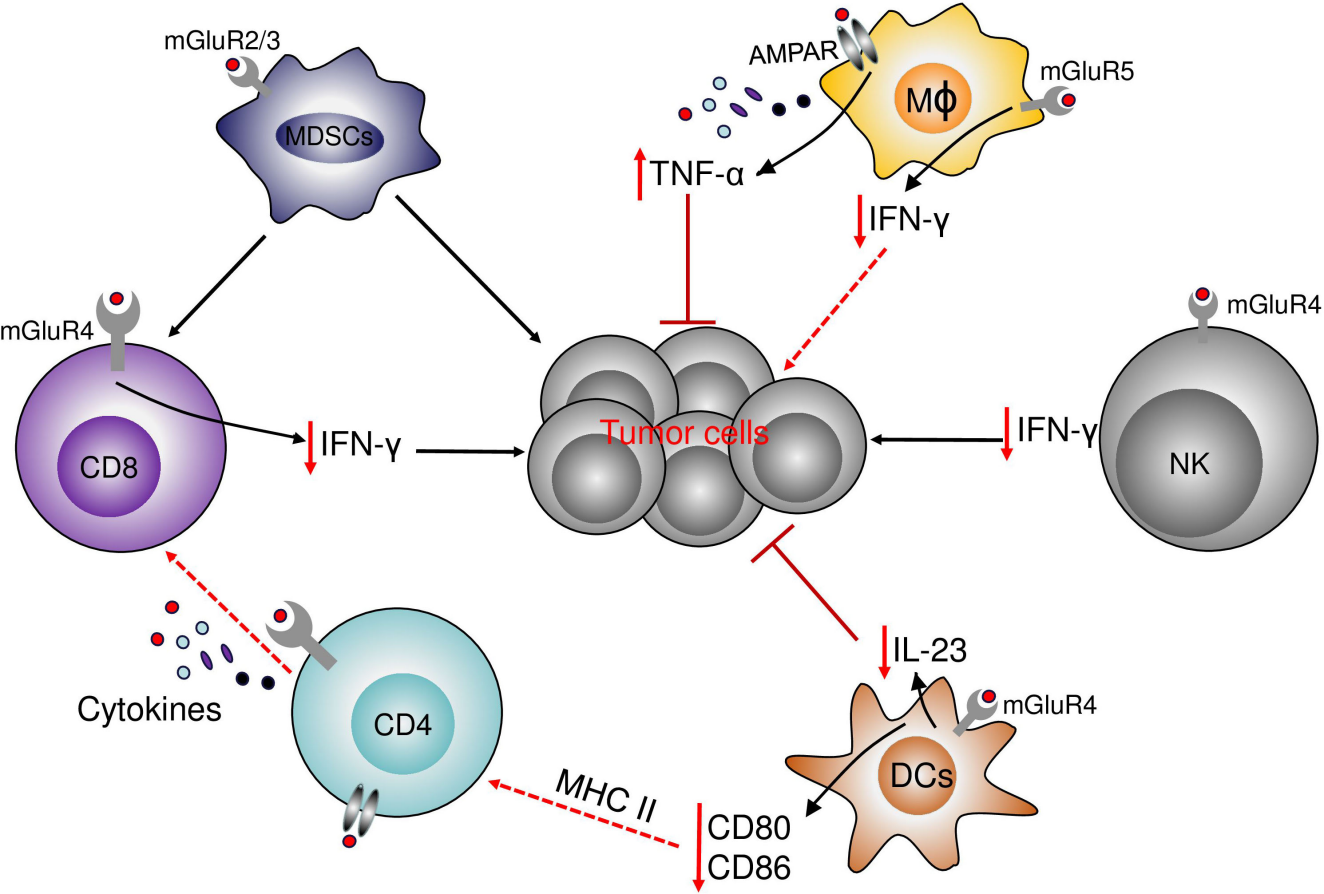
The role of glutamate receptors in immune cells. The activation of mGluR4 on the surface of DCs decreases the production of the protumor IL-23 and decreases tumor growth. mGluR4 also increases the expression of CD80 and CD86. In NK and T cells, the activation of mGluR4 decreases the antitumor immune response by the decrease in NK and T cells infiltration and the decrease in IFN-γ production. In Myeloid-derived suppressor cells (MDSCs), the activation of mGluR2/3 decrease the immune-suppressive properties of MDSCs on T cells and decreases the effects of MDSCs in cancer cells. In macrophages, the activation of mGluR5 increases the level of PPAR-γ and a shift from M1 to M2 phenotype and the decrease in IFN-γ production, while the activation of NMDAR increases the production of TNF-α.

Glutamate is particularly important for the metabolism of immune cells. It is, reasonable to hypothesize that, the glutamate receptor can regulate the response of immune cells and mediate the effective antitumor immune response (**Figure 5**).

#### 5.1.1 mGluR5 and immune cells

Macrophages that are an important component of TME, can be polarized into pro-inflammatory M1 or anti-inflammatory M2 macrophages. The polarization of macrophages can be determined by several environmental factors including cytokines and metabolites such as glutamate. The regulation of macrophage metabolism is important for the effectiveness of the antitumor immune response. mGluRs are expressed by macrophages. In microglia stimulated by LPS, the activation of mGluR5 by the agonist was associated with the reduced activation of microglia and the decrease in pro-inflammatory mediators such as INF-γ. In RAW264.7, the overexpression of mGluR5 increases the production of IL-10, indicating that the activation of mGluR5 can induce the polarization of macrophages toward the M2 type (116). The overexpression of mGluR5 was correlated with increases in the level of PPAR-γ. Activation of PPAR-γ induces a shift from the M1 phenotype to the M2 phenotype in macrophages (117). This result suggests that the polarization of macrophages by the stimulation of mGluR5 might be through PPAR-dependent transcription systems (116). Although the role of mGluRs has been investigated in macrophage in inflammatory diseases such as multiple sclerosis, the role of mGluRs has not been investigated in macrophage in a cancer model. Macrophages are important elements in the immune response against cancer, illustrated by the most important population of immune cells in the TME represented by tumor-associated macrophages (TAM). Also, mGluRs are expressed in myeloid cells, including macrophages, therefore it is probable that mGluRs regulate the response of macrophages in cancer. The regulation of the inflammatory response by macrophages and the regulation of the balance between M1 and M2 macrophages would be a benefit for the understanding of the mechanisms of the immunosuppressives phenotype in the tumor.

#### 5.1.2 mGluR2/3 and immune cells

Myeloid-derived suppressor cells (MDSCs) are immunosuppressive myeloid cells that can suppress the antitumor immune response and promote tumor growth in patients with cancer. MDSCs can suppress the response of T cells and promote the growth of tumor cells (83). The relationship between MDSCs and glutamate receptors was investigated by Morikawa et al. with the use of mGluR2/3 antagonist LY341495. The inhibition of mGluR2/3 by the antagonist resulted in the attenuation of the immunosuppressive activity of MDSCs and decreased the growth of B16F10 cells (83), probably by the increase of the antitumor immune response. These results indicate that mGluR2/3 can promote tumor growth and progression by inducing MDSCs or enhancing their immunosuppressive function. The expression of mGluR2/3 was detected in MDSCs by flow cytometry. Moreover, the expression of mGluR2/3 was detected at a low level in MDSCs and the expression was not confirmed by other methods, therefore, further experiments are needed to confirm and validate that the low-level expression of mGluR2/3 in MDSCs cells is critical.

#### 5.1.3 mGluR4 and immune cells

The activation of mGluR4 can regulate the Th17 immune response. mGluR4 is expressed by dendritic cells (DCs) (118, 119). As antigen-presenting cells, DCs are very important in the communication between innate and adaptive immune systems. DCs express co-stimulatory ligands, and cytokines to drive the T-cell differentiation. DCs govern the differentiation of naïve CD4^+^ T helper (Th) cells into effector cells by the production of different types of cytokines. The production of TGF-β, IL-6, and IL-23 induces the differentiation of naive T cells into Th17 cells, which are involved in immunity against fungi and extracellular bacteria (120). The overexpression of mGluR4 in DCs was correlated with the decreased expression of CD80 and CD86, whereas the knockdown of mGluR4 did not influence the expression of CD80 and CD86, but increased the production of IL-17A by bone-marrow-derived dendritic cells (BMDC) together with the increased production of IL-6 and IL-23 (118). As previously reported, mGluR4 could regulate cAMP leading to the regulation of the response of DCs. In melanocytes, Th17 cytokines can regulate the microphthalmia-associated transcription factor (MITF). Treatment of DCs with Th17-related cytokines decreases the expression of MIFT and decreases melanin production in B16F10 cells (118). The regulation of DCs by mGluR4 could exert beneficial effects on the antitumor immune response. Curcumin with its antioxidant, anti-inflammatory, and anti-cancerogenic properties can stimulate BMDCs through mGluR4. The stimulation of mGluR4 by curcumin significantly reduces the production of IL-6 and IL-23 by DCs and induces the suppression of Th17 cell differentiation (119). These results provide insights into the mechanism by that curcumin regulates DC-mediate immune responses in disease settings.

Our previous study demonstrated that mGluR4 played a role in osteosarcoma *via* a non-cell-autonomous mechanism. Using an irradiation-induced osteosarcoma model, we found that Grm4^-/-^ mice were more susceptible to developing tumors compared with WT mice, and treatment with an agonist of mGluR4 efficiently suppressed osteosarcoma progression. A previous study by Kansara M et al. (121) reported that mGluR4 regulated cytokine production, including IL-12 and IL-23 which played important roles in regulating tumor immunity. In line with this, IL-23 promotes osteosarcoma progression in our models as IL-23 deficient or WT mice treated with anti-IL-23 neutralizing antibody-suppressed tumor progression. We found that mGluR4 selectively suppressed IL-23 production rather than IL-12 in tumor myeloid cells. In this work, we reported that mGluR4 was expressed by CD45^+^CD11c^+^MHC^+^ myeloid cells, and mGluR4 was also expressed in spleen and bone marrow-derived dendritic cells and macrophages (120, 121). The expression of mGluR4 was detected at a very low level in CD4^+^ T cells in the mouse model of osteosarcomas (121), but not in CD8 T and NK cells.

Different from our findings in the osteosarcoma model, a recent study reported the immune-promoting role of mGluR4 in other tumor models. Using the single-cell RNA sequence (scRNA-seq) and flow cytometry, Wan et al. showed that mGluR4 was expressed in several types of immune cells, including CD4^+^ T cells, CD8^+^ T cells, NK cells in B16F10, 3LL, and MC38 mice tumor models (122), although this has not been validated elsewhere. Grm4^-/-^ mice significantly suppressed B16F10, 3LL, and MC38 tumor growth (122). This effect was associated with the increased infiltration and proliferation of NK cells and CD8^+^ T cells. The deletion of Grm4 enhances the functionality in the sub-cluster of CD8-C7-Itgb1 which is highly active in producing IFN-γ (122), indicating that mGluR4 also regulates the function of CD8 T cells in the TME. mGluR4 can negatively regulate the expression of cAMP (122, 123). The activation of cAMP by mGluR4 knockout in the tumor was associated with the increase in antitumor immune response played by CD8^+^ T and NK cells, with the increased levels of pCREB and IFNGR1 in Grm4^−/−^ CD8^+^ T cells. It is interesting to note that, the inhibition of Grm4 in wild-type by the selective antagonist (RS)-α-Methylserine-O-phosphate (MSOP), induces the same effect as observed in Grm4^−/−^ mice indicating the crucial role played by mGluR4 in the regulation of the antitumor immune response (122). Several factors can explain the different roles of mGluR4 in tumor models. Firstly the type of tumor cell used can regulate some immune cells in the TME. In the osteosarcoma model, the stimulation of marrow-derived DCs with osteosarcoma cell conditioned media (OS-CM) increased the expression of IL-23 suggesting that osteosarcoma cells can regulate the tumor microenvironment, which was not the case with B16F10, 3LL, and MC38 cells (121). Secondly, the type of immune cells investigated can lead to a difference in the immune response. Thus, the knockout of mGluR4 in DCs increases the expression of IL-23 which is a pro-tumor cytokine in the osteosarcoma model (121), while the deletion of mGluR4 in CD4, CD8 T, and NK cells improves the immune response and decreases the tumor growth (122). All this together indicates that the role of mGluR4 in immune cells in a tumor setting is controversial and context-dependent.

### 5.2 The inotropic glutamate receptors and the immune cells

Both NMDAR and AMPAR which are sub-type of iGluR are expressed by immune cells such as T cells (124, 125) and macrophages (126) among others.

#### 5.2.1 NMDAR and AMPAR and immune cells

Few studies have investigated the role played by iGluR in the regulation of immune cells in TME, and most of the studies have investigated the role of iGluRs in immune cells during auto-immune diseases such as encephalomyelitis. Nevertheless, the role of iGluRs in immune cells, in general, has attracted the attention of some researchers. In the macrophage cell line (RAW264.7), the production of pro-inflammatory TNF-α was shown to be regulated by the activation of AMPAR. The AMPAR is expressed by RAW264.7 and the activation of AMPAR was correlated with the increase in the production of TNF-α and the activation of nuclear factor (NF)-κB. The inhibition of AMPAR by the antagonist CNQX abolished the production of TNF-α. The blockage of NF-κB by pyrrolidine dithiocarbamate (PDTC), also decreases the production of TNF-α (126). The role of AMPAR in primary macrophages remains to be investigated. Although the NMDAR is crucial for the communication between the neurons in the brain, the expression of NMDAR has not been detected in microglia so far, and the possible explanation is still unknown.

The role of AMPAR in the adhesion of lymphocytes was demonstrated several years ago by the use of agonists (124). At a normal physiological concentration, glutamate through iGluR in particular AMPAR induces adhesion of normal resting human T cells to two major glycoproteins of the extracellular matrix (ECM): fibronectin and laminin. The adhesion of T cells is important for the migration, extravasation, and homing of T cells from the blood and lymphatic system into solid organs and various tissues (124). A similar result was observed in Jurkat T-leukemia where the stimulation of AMPAR by glutamate induces the adhesion of the human T-cell leukemia line (Jurkat) to fibronectin (127), and increased *in vivo* engraftment into the liver and chorioallantoic membrane of a chick embryo, allowing the spread of human T-cell leukemia and T-cell lymphoma (124). The activation of AMPAR in T cells is also important in increasing the chemotactic migration of naïve and resting normal human T cells toward the crucial chemokine CXCL12/SDF-1 (124). The use of MK-801, an NMDAR antagonist, was shown to inhibit the increase in Ca^2+^ influx in T cells, showing the role of this receptor in the control of Ca^2+^ influx in lymphocytes. The secretion of inflammatory cytokines is important in the antitumor immune response provided by T cells. The stimulation of NMDAR by glutamate suppressed the production of inflammatory cytokines such as suppressed IFN-γ secretion by IL-2-activated T cells. Some studies have also shown that glutamate can increase the production of inflammatory cytokines in normal physiological conditions (125). Of note, the net effect readout of glutamate stimulation should be the integration of various glutamate receptors and glutamate metabolism in the cells. Therefore, experiments based on specific antagonists, agonists against the receptors, and blocking antibodies should be employed for the interpretation of the observations.

The activation of NMDAR by ketamine, a group of rapid-acting antidepressants, leads to the activity-dependent release of brain-derived neurotrophic factor, activation of the mTOR pathway in the CNS, and increased protein synthesis and synaptic strength (128). Depressive disorders are associated with the activation of monocytes/macrophages releasing inflammatory cytokines such as IL-6 and IL-12. The treatment of macrophages with Ketamine induced the polarization of macrophages into M2c-like macrophages by increasing CD163 and Mer tyrosine kinase (MERTK) expression, resulting in the reduction of macrophage activation markers, especially CD80 and HLA-DR (128). Interestingly, the treatment of macrophages with MK-801, an NMDAR antagonist, showed similar results to that observed with ketamine, suggesting that ketamine and MK-801 could induce beneficial effects by the activation of NMDAR in depressive disorders (128). In contrast, the immune modulation of cancer requires an increase in the pro-inflammatory response that could have a beneficial effect in the reduction of tumor growth. Moreover, M2 macrophages promote tumor growth, therefore, the use of an NMDAR agonist that can have the opposite effect of the antagonist could improve the antitumor response of macrophages.

## 6 Crosstalk between inotropic and metabotropic glutamate receptors

iGluRs and mGluRs respond primarily to glutamate as ligands. Similarly, these receptors are expressed simultaneously in some cells. Because of that, the crosstalk between iGluR and mGluRs can be possible during their signaling mechanism. Crosstalk between iGluRs and mGluRs can occur at different levels

### 6.1 Direct interaction between iGluR and mGluR

GluRs are transmembrane proteins that possess extracellular domains and intracellular C-terminal extensions, it’s, therefore, possible for these receptors to interact. Although there is little evidence of interaction between iGluRs and mGluRs receptors, a dynamic interaction between the C-terminal domain (CTD) of mGluR5 and NMDAR (GluN1a/2B) receptors have been reported in the human embryonic kidney (HEK) 293 cells using bioluminescence resonance energy transfer (129). Because of this crosstalk, the mGluR5 receptor decreased the NMDAR current, and reciprocally, the NMDAR strongly reduced the ability of the mGluR5 receptor to release intracellular calcium (129).

### 6.2 Crosstalk through common downstream signaling

iGluRs and mGluRs are involved in the activation of many of the same downstream signaling pathways, where crosstalk can occur. The activation of NMDAR in particular induces the influx of extracellular Ca^2+^, which acts as a second messenger in the regulation of the different processes. Similarly, the group I mGluRs increase the intracellular Ca^2+^ concentration *via* a classical Gq-mediated mechanism that triggers release from intracellular stores through IP3 receptors (130). Therefore, mGluRs (mGluR5) can induce the activation or the inhibition of voltage-gated Ca^2+^ channels. On the other hand, the influx of Ca^2+^ can be controlled by NMDAR. The activation of mGluR5 and NMDAR can induce a convergence point for regulating Ca^2+^.

Another convergence point involving the iGluRs and mGluRs is the activation of Ca^2+^/calmodulin-dependent protein kinase II (CaMKII). Indeed, the influx of Ca^2+^ induces the phosphorylation of CaMKII at Thr286, which enhances CaMKII binding to NMDAR (GluN2B) (131). It has also been shown that CaMKII can induce the alteration of mGluR1 and mGluR5 function by binding to their intracellular domains, leading to possible crosstalk between iGluRs and mGluRs.

As mGluRs belong to the family C G-protein-coupled receptors (GPCR), they primarily activate either Gq or Gi/o pathways. Some evidence has suggested that iGluRs can also activate G proteins to produce metabotropic signaling (132). The activation of AMPAR and KA induces the Gi/o-like signals and PLC/PKC activation, respectively, however, little is known about the mechanisms by which these iGluRs could activate mGluRs downstream (133, 134).

To date, there are no studies investigating the crosstalk between iGluRs and mGluRs in cancer and immune cells within the TME. As these receptors are expressed simultaneously in some cancer and immune cells, their possible crosstalk may open new perspectives in the regulation of the TME by GluRs. The use of combined therapy that can inhibit or activate different downstream signaling of iGluRs and mGluRs in cancer and immune cells could enhance the effectiveness of the antitumor response

## 7 Cooperation of homomeric and heteromeric metabotropic glutamate receptors in the TME

mGluRs belong to the family C G-protein-coupled receptors (GPCR), and the activation of mGluRs by extracellular glutamate induces the activation of the intracellular heterotrimeric G proteins. In general, mGluRs are dimer proteins, and their activation requires the formation of dimer complexes before becoming functional receptors. In physiological conditions, all mGluRs are capable of forming homodimeric complexes. This complex is formed by the assembly of two identical proteins (mGluR2-mGluR2). The pharmacological properties of each mGluRs have attracted several investigations for the modulation of neuronal disorders or cancer therapy, and the discovery of the potential that the homodimers can offer in the modulation of different pathologies led to the exciting possibility of heterodimerization. It has recently been demonstrated that mGluRs can indeed form heterodimers with unique pharmacological and functional properties (135, 136). Heterodimerization represents the formation of covalently linked dimer complexes composed of two different members of the mGluR family. The heterodimer assembly requires the co-existence of the two receptors on the cell surface of the same cell. On the other hand, some mGluRs have some preferences for some heterodimers compared to others. mGluR1 has been shown to have a preference for heterodimer with mGluR5. Interestingly, in mGluR1/5, the binding of the agonist to one of the subunits was necessary for receptor activation (137), while the activation of mGluR1 homodimer required the binding of the ligand to both subunits (137). The preference for heterodimer formation between mGluR2/4 was described in rat neurons. The pharmacological activation of mGluR2 and mGluR4 by their selective agonists DCG-IV or L-AP4 separately showed a weak activation, while the full activation was observed by their combined application (138). Interestingly, the inhibition by a semi-selective orthosteric antagonist LY341495 at concentrations that only inhibit mGluR2 inhibited the receptor partially, whereas complete inhibition is achieved with a concentration that inhibits both mGluR2 and mGluR4 (139). The heterodimerization between mGluR2 and mGluR7 was reported to enhance the biophysical properties of the heterodimer compared to mGluR7 homodimers. The reason for this phenomenon is not understood (135). In the frontal cortex, there is a prominence in the mGluR2/3 heterodimerization than mGluR3 homodimerization. The high level of receptor expression and the enhanced propensity for heterodimerization of one of the subunits are necessary for the shift of homo- to heterodimers (140).

The large majority of studies related to mGluRs heterodimerization were done in the brain. There is no data about the potential implication of the mGluRs heterodimers in immune cells and cancer in the TME. As mentioned above the activation of mGluRs homodimers induce the activation of the downstream signaling. The capacity of the heterodimers to increase the biophysical response after mGluRs desensitized could be fundamental in enhancing the antitumor immune response. Indeed, if the binding of the agonist to the same subunit of the homodimer can induce a signal, the binding of the agonist to two subunits forming the heterodimer could increase the signal by the combination of the two subunits. Similarly, in some cancer cells where the activation of mGluRs is tumor-promoting such as mGluR8, the heterodimerization of the receptor with another receptor could enhance the efficacity of the antagonist in the inhibition of tumor growth. The development of a potential target drug for the modulation of the receptor, by the study of the pharmacodynamic role of the heterodimer, especially for cancer therapy could be an important element to consider in future research.

## 8 Summary

In this manuscript, by a systematic review of available data, we reported the physiopathological role of glutamate receptors in the regulation of cancer and immune cells and explored the potential implications for a future therapeutic strategy in cancer. The available data analyzed in this review showed that several transporters are involved in the regulation of glutamate availability, which may contribute to different metabolic processes that can regulate tumor growth and the immune response in the TME. Glutamate is also involved in the activation of glutamate receptors that are expressed by tumor and immune cells within the TME (5, 14), and, the expression of GluRs varies depending on the type of tissue and the type of cells. Different studies have also reported that the expression of GluRs in a specific type of tissue or cell seems to be proportional to the role played by the receptor. Furthermore, the activation and the expression, of GluRs in cancer and immune cells in the TME, may play a key role in the regulation of tumor growth (5). In cancer cells, different signaling pathways are activated by GluRs resulting in the regulation of cancer cell growth which is very important in cancer therapy. As a component of the TME, immune cells are very important in the antitumor immune response, therefore, the activation of GluRs in immune cells may have beneficial effects in the regulation of antitumor immune response in some tumor types (122). Although some advanced have been done in the understanding of the role played by GluRs in cancer, the gap between what has been done and what needs to be done is still big. For instance, several drugs that can modulate the response of GluRs have been used in animal experiments with satisfactory results, however, there is still a lack of very solid data on the use of these drugs in a clinical trial. Furthermore, the cooperation of different GluRs in the regulation of cancer and immune cells, as well as the type of mutation in GluRs that can trigger cancer development is poorly understood. Lastly, as a neurotransmitter, the regulation of GluRs by CNS needs further investigation.

The CNS that orchestrates and controls the response of effector cells might have an important role in the regulation of GluRs in immune cells and effector cells within the TME. For now, most of the studies have reported the role of GluRs in peripheral organs and immune cells. Therefore, the role of the afferent neurons in the regulation of immune response in the tumor could be an important element to consider for an effective antitumor immune response. Recently in 2022, Kavita Vats et al. (141) opened the way for the investigation of the potential role of the sympathetic nervous system (SNS) in the regulation of the immune cells and the TME. They revealed that the sensory innervation limited the activation of effective antitumor immune responses, while the sensory ablation significantly improved leukocyte recruitment into subcutaneous tumors, leading to the decreased presence of lymphoid and myeloid immunosuppressive cells and increased activation of T-effector cells within the TME. Interestingly, their study demonstrates that the SNS through the afferent neurons negatively regulates the antitumor immune response and tumor growth. However, little is known about the relationship between the SNS and GluRs in the regulation of antitumor immune response and the regulation of tumor growth in the periphery organs and tissues. The mechanism by which the SNS and afferent neurons regulate the TME through GluRs could be a step forward for the potential strategy to improve the antitumor immune response and reduce tumor growth.

## Author contributions

JY conceived the idea. JY, JH, and SK wrote the manuscript and SK was the major contributor in writing the manuscript. JY, SK, and SS designed the figures. JH, XJ and GS revised the manuscript. R-XT and K-YZ revised the manuscript and provided fundamental ideas. All authors contributed to the article and approved the submitted version.
